# Plant genomic resources at National Genomics Data Center: assisting in data-driven breeding applications

**DOI:** 10.1007/s42994-023-00134-4

**Published:** 2024-02-02

**Authors:** Dongmei Tian, Tianyi Xu, Hailong Kang, Hong Luo, Yanqing Wang, Meili Chen, Rujiao Li, Lina Ma, Zhonghuang Wang, Lili Hao, Bixia Tang, Dong Zou, Jingfa Xiao, Wenming Zhao, Yiming Bao, Zhang Zhang, Shuhui Song

**Affiliations:** 1 National Genomics Data Center, Beijing Institute of Genomics, Chinese Academy of Sciences & China National Center for Bioinformation, Beijing, 100101 China; 2 CAS Key Laboratory of Genome Sciences and Information, Beijing Institute of Genomics, Chinese Academy of Sciences & China National Center for Bioinformation, Beijing, 100101 China; 3 University of Chinese Academy of Sciences, Beijing, 100049 China

**Keywords:** Plant-omics data, Data repositories, Data integration, Knowledgebase, Plant genomics

## Abstract

Genomic data serve as an invaluable resource for unraveling the intricacies of the higher plant systems, including the constituent elements within and among species. Through various efforts in genomic data archiving, integrative analysis and value-added curation, the National Genomics Data Center (NGDC), which is a part of the China National Center for Bioinformation (CNCB), has successfully established and currently maintains a vast amount of database resources. This dedicated initiative of the NGDC facilitates a data-rich ecosystem that greatly strengthens and supports genomic research efforts. Here, we present a comprehensive overview of central repositories dedicated to archiving, presenting, and sharing plant omics data, introduce knowledgebases focused on variants or gene-based functional insights, highlight species-specific multiple omics database resources, and briefly review the online application tools. We intend that this review can be used as a guide map for plant researchers wishing to select effective data resources from the NGDC for their specific areas of study.

**Supplementary Information:**

The online version contains supplementary material available at 10.1007/s42994-023-00134-4.

## Introduction

In the face of escalating global challenges such as continued population growth, extreme climate change, and water scarcity, the attainment of food and nutrition security can be achieved by greatly accelerating improved crop breeding (Hickey et al. 2019). In recent years, internationally proposed concepts such as ‘Breeding 4.0’ (Wallace et al. 2018) and ‘5G Breeding’ (Varshney et al. 2020) have gained prominence, with their successful implementation relying on the substantial support from extensive omics data. Taking the model organism *Arabidopsis thaliana* as an example, researchers can comprehensively explore various fundamental biological phenomena through the extensive resources provided by the TAIR database (Lamesch et al. 2012).

The advancement of high-throughput sequencing technology has given rise to the rapid expansion of big data agricultural data. Notably, both the National Center for Biotechnology Information (NCBI) and the European Bioinformatics Institute (EBI) have established a number of database resources dedicated to the storage and management of plant-related data. These comprehensive resources encompass SRA (Leinonen et al. 2011) and ENA (Burgin et al. 2023) for raw sequencing data archiving, RefSeq (O’Leary et al. 2016) and Ensembl Genome (Kersey et al. 2010) for the housing of reference genome sequences, GEO (Barrett et al. 2012) and Expression Atlas (Moreno et al. 2022) for the dissemination of gene expression data, EVA (Cezard et al. 2022) for cataloguing plant genome variation, and Gramene (Tello-Ruiz et al. 2016) for comparative plant genomics and complex pathway analysis. In addition, Phytozome (Goodstein et al. 2012), a comparative genomics research portal developed by the Department of Energy’s Joint Genome Institute, now hosts 318 assembled and annotated genomes in its latest v13 release. At the same time, several species-specific multi-omics resource platforms integrating multi-dimensional data have emerged around the world to gain a comprehensive understanding of plant trait response mechanisms. Prominent examples such as RFGB (Wang et al. 2019) and MBKbase (Peng et al. 2020) for rice, BnIR (Yang et al. 2023b) for rapeseed, MaizeGDB (Portwood et al. 2019) for maize, CottonMD (Yang et al. 2023a) for cotton, and others, allow comprehensive exploration of molecular intricacies and variations across diverse levels, including the genome, epigenome, transcriptome, proteome, metabolome, and phenome (Yang et al. 2023b).

**Fig. 1:**
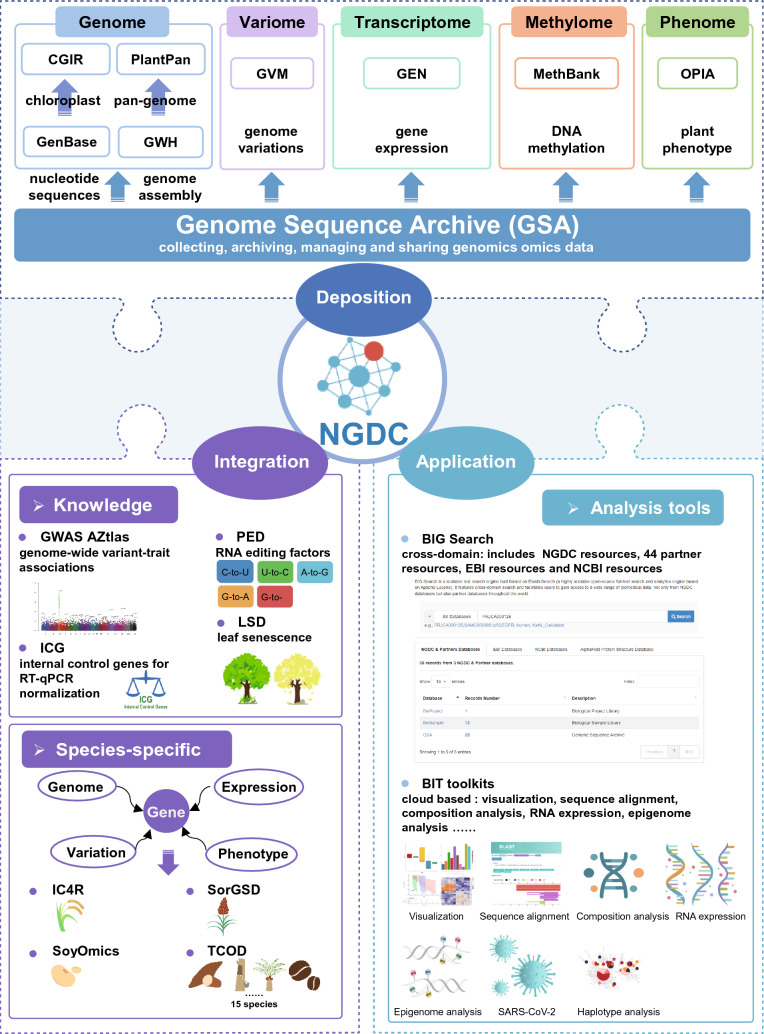
Overview of database resources and application tools for plants in the CNCB-NGDC

As a prominent player in both agriculture and genomics, China produces vast amounts of data, but faces challenges such as scattered data distribution, inadequate data management, and limited data reusability. The National Genomics Data Center (NGDC), part of the China National Center for Bioinformation (CNCB), was established to pioneer the development of a comprehensive national biological big data management system in China. NGDC is dedicated to advancing the life and health sciences by providing open access to a range of data resources and services to support of global research activities on big data archiving, storage, management and public sharing as well as multidisciplinary data-driven research (BIG Data Center Members 2018, 2019; CNCB-NGDC Members and Partners 2020, 2021, 2022, 2023). Now, a comprehensive plant data resources were available, which primarily comprises three categories: (1) multi-omics databases, including GSA (Chen et al. 2021c), GWH (Chen et al. 2021b), CGIR(Hua et al. 2022), GVM (Li et al. 2021), PlantPan, GEN (Zhang et al. 2022), MethBank (Zhang et al. 2023) and OPIA (Cao et al. 2023); (2) variants or gene-based functional knowledgebases, including GWAS Atlas (Liu et al. 2023a), PED (Li et al. 2019), LSD (Li et al. 2020) and ICG (Sang et al. 2018); and (3) species-specific integrated resources, including IC4R (Sang et al. 2020), RED (Xia et al. 2017), SoyOmics (Liu et al. 2023b), SorgSD (Liu et al. 2021) and TCOD (Kang et al. 2023). Additionally, NGDC has also developed several tools for plant data mining and analysis. These invaluable resources and tools provide robust support for China’s agricultural research during its transition from breeding 2.0 to 4.0. In this review, we give a summary illustration of the plant-related resources in NGDC (Fig. 1) and appeal to the plant research community to make full use of them.

## Multi-omics data repositories

### Repositories for genomic and phenotypic data archiving

Benefiting from the development and application of second/third-generation sequencing and high-throughput plant phenotype acquisition technologies, enormous amounts of omics data are massively generated and have revolutionized plant genetic research and crop improvement strategies (Bellare et al. 2018; Shendure and Ji 2008). Therefore, the development of repositories to collect and organize multi-omics data is of great importance and crucial for the long-term preservation and archiving of these genomic sequence data. The NGDC has established a number of functional databases, listed in Table 1, to archive and share plant omics data. Several core archival repositories support the genomic data submission, archiving, preservation, and sharing. Among them, Genome Sequence Archive (GSA; https://bigd.big.ac.cn/gsa) is a public data repository for archiving raw sequence reads, Genome Warehouse (GWH; https://bigd.big.ac.cn/gwh) is a public resource archiving genome-scale data of a wide range of species, GenBase (https://ngdc.cncb.ac.cn/genbase) is an open-access data repository dedicated for archiving, searching, and sharing nucleotide sequences, while Genome Variation Map (GVM; https://bigd.big.ac.cn/gvm) is a public repository of genome variation data, and Open Plant Image Archive (OPIA; https://ngdc.cncb.ac.cn/opia/) an open archive of plant images and image-based phenotypic traits (i-traits) data. All the five databases accept worldwide data submissions, provide data curation and quality control for all submitted data, and offer free open data sharing services for all publicly available data.

**Table 1:** Database resources of plants in the CNCB-NGDC

Type	Database	Web link	Functionality	Data volume
Repositories	Genome Sequence Archive (GSA)	https://ngdc.cncb.ac.cn/gsa	Data repository for collecting, archiving, managing and sharing raw sequence data generated from different platforms	1850 plant species 154,749 experiments 173,258 runs ~ 4500 TB volume
	Genome Warehouse (GWH)	https://ngdc.cncb.ac.cn/gwh	Centralized resource housing genome-scale data for a wide range of species and delivering a series of web services for genome data submission, storage, release and sharing	1423 plant species 10,594 assemblies
	GenBase	https://ngdc.cncb.ac.cn/genbase	Accepts user submissions (mRNA, genomic DNAs, ncRNA, or small genomes such as organelles, viruses, plasmids, phages from any organism) and integrates data from INSDC	1085 proteins sequences 1024 nucleotide sequences
	Chloroplast Genome Information Resource (CGIR)	https://ngdc.cncb.ac.cn/cgir	Provides curated resource of chloroplast genome information, dedicating to the integration, annotation and standardization of chloroplast genomes, genes, simple sequence repeats (SSR), and DNA signature sequences (DSS)	16,435 plant species 5918 featured plants 29,069 genomes
	Genome Variation Map (GVM)	https://ngdc.cncb.ac.cn/gvm	Public repository of genome variations, providing single nucleotide polymorphisms (SNPs) and small insertions & deletions (InDels) presentation and variome data archiving	30 plant species 72 projects 34,643 samples
	Gene Expression Nebulas (GEN)	https://ngdc.cncb.ac.cn/gen	Provide data portal of transcriptomic profiles under various conditions derived entirely from bulk and single-cell RNA-Seq data analysis in multiple species	10 plant species 1867 samples 22,215 single-cells
	Methylation Bank (MethBank)	https://ngdc.cncb.ac.cn/methbank	Banks of integrated DNA methylomes across a variety of species. Providing whole genome single-base methylome and manually curate knowledge of both featured differentially methylated genes	7 plant species 236 tissues/cell lines 1449 single-base resolution methylomes
	Plant pan-genome (PlantPan)	https://ngdc.cncb.ac.cn/plantpan	Encompasses pan-genome analysis results from multiple plant species, provides gene-based and graph-based pan-genome for each species, along with detailed gene functions, 13 types of genomic variations, and genome synteny	11 plant species 195 genomes 9,127,208 genes 413,000,124 genomic variations 3,345,098 genome synteny
	Open Plant Image Archive (OPIA)	https://ngdc.cncb.ac.cn/opia	An archive database of plant images and related phenotypic data from high-throughput phenotyping platforms for a diversity of species	11 plant species 56 datasets 566,225 images 56 i-traits
Knowledge databases	GWAS Atlas	https://ngdc.cncb.ac.cn/gwas/	Manually curated resource of genome-wide genotype-phenotype (G2P) associations for a wide range of species	10 plant species 269,138 association 630 publications
	Plant Editosome Database (PED)	https://ngdc.cncb.ac.cn/ped	Provides RNA editing factors, RNA editing events of targeted organelle genes, interactions between editing factors and events in multiple species, biological functional effects of editing factors in regulating plant phenotypes and the corresponding experimental detailed information	1618 plant species 144 editing factors 203 edited genes 25,164 editing evens 137 publications
	Internal Control Genes (ICG)	https://ngdc.cncb.ac.cn/icg	Provides a comprehensive collection of high-quality experimentally verified internal control genes and their application scenarios for both model and non-model organisms	278 plant species 340 studies 1216 genes
	Leaf Senescence Database (LSD)	https://ngdc.cncb.ac.cn/lsd/	Provides senescence associated genes, mutants, phenotypes and literature references	86 plant species 31,214 genes 1037 mutants
Species-specific resources	Information Commons for Rice (IC4R)	http://ic4r.org/	A curated database providing rice genome sequences, updating rice gene annotations and integrating multiple omics data through community-contributed modules	56,221 protein-coding genes 80,038 protein-coding transcripts 6259 long non-coding RNAs 4373 circular RNAs 1503 RNA-Seq datasets
	Rice Expression Database (RED)	http://expression.ic4r.org	Provides gene expression profiles derived entirely from RNA-Seq data analysis on tissues spanning an entire range of rice growth stages and covering a wide variety of biotic and abiotic treatments	9 tissues 24 projects 284 experiments
	SoyOmics	https://ngdc.cncb.ac.cn/soyomics	Provides a wide variety of soybean multi-omics data, encompassing assembled genomes, graph pan-genomes, resequencing data, phenotypic information from representative germplasms, transcriptomic and epigenomic data from different tissues, organs, and accessions, as well as knowledge of quantitative trait locus and genome-wide association study	Genome of 27 cultivars variome of ~ 3000 soybean germplasms transcriptome of 28 tissues phenome of 115 traits homology and synteny of 28 genomes
	SorgSD	https://ngdc.cncb.ac.cn/sorgsd	Provides a wealth of sorghum-related information, including genome, variations, phenotypes, panicle images, online resources and critical references	289 sorghum accessions 39,547,621 variations 289 phenotypes
	Tropical Crop Omics Database (TCOD)	https://ngdc.cncb.ac.cn/tcod	Provides genome sequences, gene function annotations, cross-species homology relationships, genome variations, gene expression and germplasm resource descriptions for 15 tropical crops	15 plant species 34 genome assemblies 1,255,044 genes 282,436,992 variants 88 expressions 13,381 cultivars

As of August 2023, GSA has archived a total of 154,749 experiments, 173,258 runs, and a total of ~ 4500 terabytes of raw sequencing data from 1850 plant species. Of these, 104,871 experiments have been published and reported in 585 journal articles. GWH has hosted a total of 10,594 assemblies for 1423 plant species, of which 1524 assemblies are publicly available and reported in 160 journal articles. GenBase has assembled 1085 protein sequences and 1024 nucleotide sequences since its public release in April 2023. GVM has received a total of 72 data submissions involving 34,643 samples from 30 plant species, and 42 projects are publicly available and reported in 42 journal articles. OPIA has hosted 56 datasets across 11 plants, comprising a total of 566,225 images with 2,417,186 labeled instances. OPIA also has incorporated 56 i-traits of 93 rice and 105 wheat cultivars based on 18,644 individual RGB images. In addition to data submitted directly by users, these repositories have also mirrored the INSDC’s data by collecting and integrating the relevant metadata and raw data from NCBI SRA, RefSeq, GenBank, and dbSNP. All the plant genomics data archived in these repositories have a total volume of approximately ~ 5 PB, cover a wide range of species, including food crops, cash crops, forage crops, and medicinal crops (Table S1), and can be retrieved via BIG search (https://ngdc.cncb.ac.cn/search), and are publicly accessible and downloadable via FTP and HTTP. Among them, rice, wheat, maize, soybean, and sorghum are widely studied crops, as they possess the highest data volume and the most comprehensive data types. The extensive data available for these crops contribute significantly to a deeper understanding of their genetic mechanisms and facilitate advances in agricultural practices.

More importantly, these repositories have been officially recognized by publishing groups and several high-profile journals. Take GSA as an example, it has been recognized as one of the certified repositories at FAIRsharing.org and re3data.org, and therefore meets the requirement as a supported repository by Elsevier, Taylor & Francis, Wiley, and Springer Nature. Up to August 2023, there have 135 scientific journals report datasets for the NDGC’s repositories.

### Databases for genomic information visualization

Omics data broadly covers, but is not limited to, measurements of the genome, transcriptome, proteome, epigenome, and metabolome. These measurements encompass the presence (binary), characterization (variation or biological function), and/or quantification (abundance) of molecules or entities, such as genes, transcripts, proteins, metabolites, or epigenetic modification (Eicher et al. 2020). These data also provide comprehensive insights into the phenotype-driven regulation of biological pathway and in turn provide preliminary evidence to the new targets or intervention strategies in breeding (Pinu et al. 2019). To provide the genetic landscape of a species and expression or methylation profiles for a specific gene, NGDC has further developed several databases, including GVM (Li et al. 2021), CGIR (Hua et al. 2022), PlantPan (CNCB-NGDC Members and Partners 2023), GenBase (CNCB-NGDC Members and Partners 2023), and MethBank (Zhang et al. 2023), promoting analysis, mining, and application of sequencing data.

The three databases (GVM, CGIR, and PlantPan) are dedicated to different types of genetic variations at the nuclear genome level, plastid genome level, and pan-genome level, respectively. GVM has made significant efforts to collect and integrate the nuclear genome variations (SNPs and small Indels) for a wide range of plant species. Based on the extensive collection of raw sequence data from public repositories and variant identification by standardized analysis pipeline, GVM houses about ~ 592 million genome variants for 29 plant species and provides user-friendly web interfaces for data search, browsing and visualization. Each variant has been assigned a unique identifier and associated details, including variant coordinates, reference and alternative alleles, and minor allele frequencies. Moreover, GVM provides comprehensive annotations for each variant, including consequence type, variant effect, population frequency, and phenotype association, and also incorporates the functional domain information from UniProt (The UniProt Consortium 2023) and Pfam (Mistry et al. 2021). In short, GVM constructs a high-density genetic variation map for each species, and is essential important for a wide range of functional studies. As chloroplast genomes have been extensively used as fundamental tools in plant phylogenetics (Daniell et al. 2021), Chloroplast Genome Information Resource (CGIR; http://bigd.big.ac.cn/cgir) were further developed by collaborating with the Chinese Academy of Chinese Medical Sciences. CGIR contains 29,069 chloroplast genomes of 16,435 species, and develops 3 commonly used DNA markers (DNA Barcodes, simple sequence repeats, and DNA signature sequences). The DNA Barcodes were identified from 29 different loci based on an in-silico approach, which are complement to traditional DNA barcode databases (e.g., Barcode of Life Data System (Ratnasingham and Hebert 2007)). Simple sequence repeats (SSR) were identified using MISA (Thiel et al. 2003) and IMEx (Mudunuri and Nagarajaram 2007), and their associated primers were designed by Primer3 (Koressaar and Remm 2007), making CGIR far superior to other plastid SSR databases (Sablok et al. 2015). More importantly, we have newly defined a DNA signature sequence (DSS), which is a nucleotide sequence of constant length capable of detecting the presence of an organism (referred to as the target species) and distinguishing it from other species (referred to as the background species). The candidate DSSs are a species-level marker that can be used as a complement to conventional DNA markers (Hua et al. 2023). All these genetic markers make CGIR a valuable resource for researchers working on phylogenetics and chloroplast genetic engineering. Along with the development of pan-genomics, which provide valuable structure variations across species and insights for biodiversity, we further developed PlantPan (https://ngdc.cncb.ac.cn/plantpan/) to encompasses pan-genome analysis result. PlantPan now offers 195 genomes from 11 plant species and provides gene-based and graph-based pan-genome for each species, and also details 13 types of genomic variations, including gene copy number variations, structural variations, and single nucleotide polymorphisms. All these variation-related databases will enhance the utilization of plant genetic materials in molecular breeding and evolutionary studies.

To reveal functional elements from transcriptional and epigenetic perspectives, NGDC developed Gene Expression Nebulas (GEN, https://ngdc.cncb.ac.cn/gen) (Zhang et al. 2022) and Methylation Bank (MethBank, https://ngdc.cncb.ac.cn/methbank) (Zhang et al. 2023). GEN is a data portal that integrates transcriptomic profiles at both bulk and single-cell levels in various conditions across multiple species. It features a curated collection of high-quality RNA sequencing datasets by using standardized data processing pipelines and a structured curation model. Specifically, 71 datasets related to 10 plant species covering 2893 samples and 220,215 cells are systematically incorporated. For each dataset, a full range of transcriptomic profiles including gene expression, circRNA expression, alternative RNA splicing and RNA editing (if applicable) are provided. Moreover, GEN accommodates value-added gene annotations based on differential expression analysis across diverse experimental conditions and cell clusters. Methbank is a comprehensive database of whole-genome DNA methylation across a variety of species. By continuously collecting whole-genome bisulfite sequencing data, MethBank 4.0 provides users with the integration, analysis, and visualization of DNA methylomes profiles from 7 plant species. Moreover, it has incorporated expert-curated knowledge modules of featured differentially methylated genes associated with biological contexts and methylation analysis tools to cater to the needs of different users. Overall, both of them provide user-friendly web functionalities and applications for large-scale data query, retrieval, analysis, and visualization.

## Variants or gene-based functional knowledgebases

The variety and quantity of plant omics data have increased dramatically in recent years, leading to an expansion of our understanding of biological systems. This abundance of data has created new challenges and opportunities for biocurators. In NGDC, many efforts have been made to curate plant knowledgebases, including the atlas of genetic variation-phenotype associations, the systematic database of leaf senescence, the high-quality feature of plant RNA editosomes, and the catalogs of experimentally validated internal control genes. We are committed to ensuring that these databases are not only reliable and reusable, but also accessible and sustainable over the long term.

GWAS Atlas (https://ngdc.cncb.ac.cn/gwas/) (Liu et al. 2023a) is a manually curated knowledgebase of genome-wide variant-trait associations in plants and animals. In the current version of GWAS Atlas, a total of 10 plant species, 630 publications, 3125 studies, 269,138 associations, 138,295 variants, 52,802 genes and 1413 traits are curated and included. More importantly, 4581 lead SNPs and 486 experimentally validated causal variants in plants are identified and integrated. All associations and traits have been annotated and organized based on Plant Trait Ontology and Plant Phenotype and Trait Ontology, respectively. Additionally, GWAS Atlas was equipped with four online analysis tools and a submission platform, allowing researchers to perform data analysis and data submission.

To facilitate the systematic research and comparative study of leaf senescence, NGDC constructed leaf senescence database (LSD; https://ngdc.cncb.ac.cn/lsd/) (Li et al. 2020) to collect senescence-associated genes (SAGs), mutants, phenotypes and literature references. LSD 4.0 contains 31,214 genes and 1037 mutants from 86 species. Through manual curation, a wide range of information, including gene name, locus name, GenBank ID, PubMed ID, mutant, species, senescence-associated phenotype, the effect on leaf senescence and evidence are retrieved. In additional, LSD makes extensive annotations for these SAGs through computational approaches, including Gene Ontology, DNA and protein sequences, protein-protein interactions, miRNA interaction information, as well as ortholog groups. These data provide important clues for researchers to elucidate the molecular regulatory mechanisms of leaf senescence.

Plant Editosome Database (PED; https://ngdc.cncb.ac.cn/ped) (Li et al. 2019) is a curated database of plant RNA editosomes, with high-quality editosome data manually curated from published literature and organelle genome annotations. The current implementation of PED houses a total of 98 RNA editing factors and 20,836 editing events, involving 203 organelle genes and covering 1621 plant species and 1673 plant organelles. In addition, PED contains interactions between editing factors and editing events in eight model species, functional effects of editing factors in regulating plant phenotypes as well as detailed experimental evidence. PED is committed to the curation, integration and standardization of plant editosome data and thus has the great potential to help researchers conduct systematic investigations on RNA editing machinery in a variety of plant species.

The Internal Control Genes (ICG; https://ngdc.cncb.ac.cn/icg) database (Sang et al. 2018) is a well-established knowledgebase of experimentally validated internal control genes and their respective applicable scenarios for RT-qPCR normalization across a wide variety of species. ICG houses a total of 1216 high-quality verified internal control genes from 278 plant species, associated with 660 corresponding applicable scenarios. The most widely used gene is *Actin*, which has been reported to be internal control gene in 137 studies. Moreover, at the tissue level, leaf associated 410 different qPCR primers in various experimental conditions were also curated. This knowledge in ICG will help the researchers to select appropriate internal control genes for their own experiments.

## Species-specific integrated resources

Taking advantage of the accumulated data resources at NGDC, we further established several staple or economic crops specific integrated resources, including rice, soybean, sorghum and dozens of tropical crops. For each crop species, we collect its omics data in multiple dimensions and use the cross-reference index as a bridge to connect different datasets, building a comprehensive one-stop service platform for researchers to obtain systematic and comprehensive knowledge.

The Information Commons for Rice (IC4R, http://ic4r.org/) (Sang et al. 2020) is a public database that integrates multiple omics data for rice and provides high-quality annotations. In its current version, by incorporating abundant information on gene expression from the Rice Expression Database (RED, http://expression.ic4r.org/) (Xia et al. 2017), IC4R comprises rich annotation and sequence information for 56,221 protein-coding genes, 6259 long non-coding RNAs, and 4373 circular RNAs, which constitute its core resources. For each protein-coding gene, IC4R provides gene summaries, transcripts, gene expression, associated functional entries, and ontologies. Meanwhile, long non-coding RNAs include coding potential scores, while circular RNAs come with supporting back-spliced junction reads. Additionally, IC4R is equipped with four online analysis tools for knowledge mining, along with community-contributed modules that support users in contributing their knowledge to improve gene annotation.

The Sorghum Genome Science Database (SorGSD, https://ngdc.cncb.ac.cn/sorgsd/) (Liu et al. 2021) is a comprehensive platform featuring sorghum genomic variations and phenotypes. In its latest release, SorGSD presents 39,547,621 genomic variations (including 33,825,236 SNPs and 5,722,385 INDELs) derived from 289 sorghum accessions, as well as characteristic phenotypic information and panicle pictures of critical sorghum lines. Moreover, SorGSD offers three useful tools: ID Conversion, Homologue Search, and Genome Browser, and grants access to a wealth of online sorghum information and published literature, serving as an invaluable platform for in-depth research on sorghum.

The SoyOmics database (https://ngdc.cncb.ac.cn/soyomics/index) (Liu et al. 2023b) is a panoramic multidimensional omics resource of soybean, providing comprehensive knowledge and analysis tools. Through the integration of 27 de-novo assembled genomes from various soybean accessions, along with their generating pan-genomes, 550,000 large-scale structural variations, and 57,480 homologous gene groups; as well as gene expression data from 28 or 9 tissue stage samples of Williams82/ZH13 or pan-genome accessions; and an approximately 38 million SNPs and INDELs derived from 2898 re-sequenced soybean samples; and approximately 27,000 records of 115 soybean phenotypes from different years and planting regions, SoyOmics offers 6 highly interactive basic modules: Genome, Variome, Transcriptome, Phenome, Homology, and Synteny for data browsing. Besides, it also offers several commonly easy-to-use toolkits, including BLAST, easyGWAS, ExpPattern, HapSnap, VersionMap and SoyArray. In summary, SoyOmics encompasses a comprehensive integration of multi-omics datasets and holds significant advantages in multi-omics interaction, pan-genome scanning, and online analysis functionality, which will greatly benefit deep mining of soybean molecular breeding study.

The Tropical Crop Omics Database (TCOD, https://ngdc.cncb.ac.cn/tcod) (Kang et al. 2023) is a comprehensive multi-omics data platform for tropical crops. TCOD encompasses 15 tropical crops, including tropical food crops like cassava, rubber crops like rubber tree, tropical fruit trees like mango, pineapple, sugarcane, banana, litchi and longan, tropical oil crops such as oil palm and coconut, tropical spicy beverages like coffee, cocoa, vanilla and pepper, as well as tropical medicinal plants like areca. In the current release version, TCOD houses 34 chromosome-level de novo assemblies, 1,255,004 genes with functional annotations, 282,436,992 unique variants from 2048 WGS samples, 88 transcriptomic profiles from 1997 RNA-Seq samples, and 13,381 germplasm items. Furthermore, in terms of analytical capabilities, TCOD not only provided homologous gene information for cross-species omics characteristics comparison but also equipped several user-friendly online tools such as BLAST, Genome Browser, Primer Design, GO Enrichment, KEGG Enrichment, Synteny Viewer, and Homolog Finder, facilitating efficient data mining and visualization.

## Application tools

**Fig. 2:**
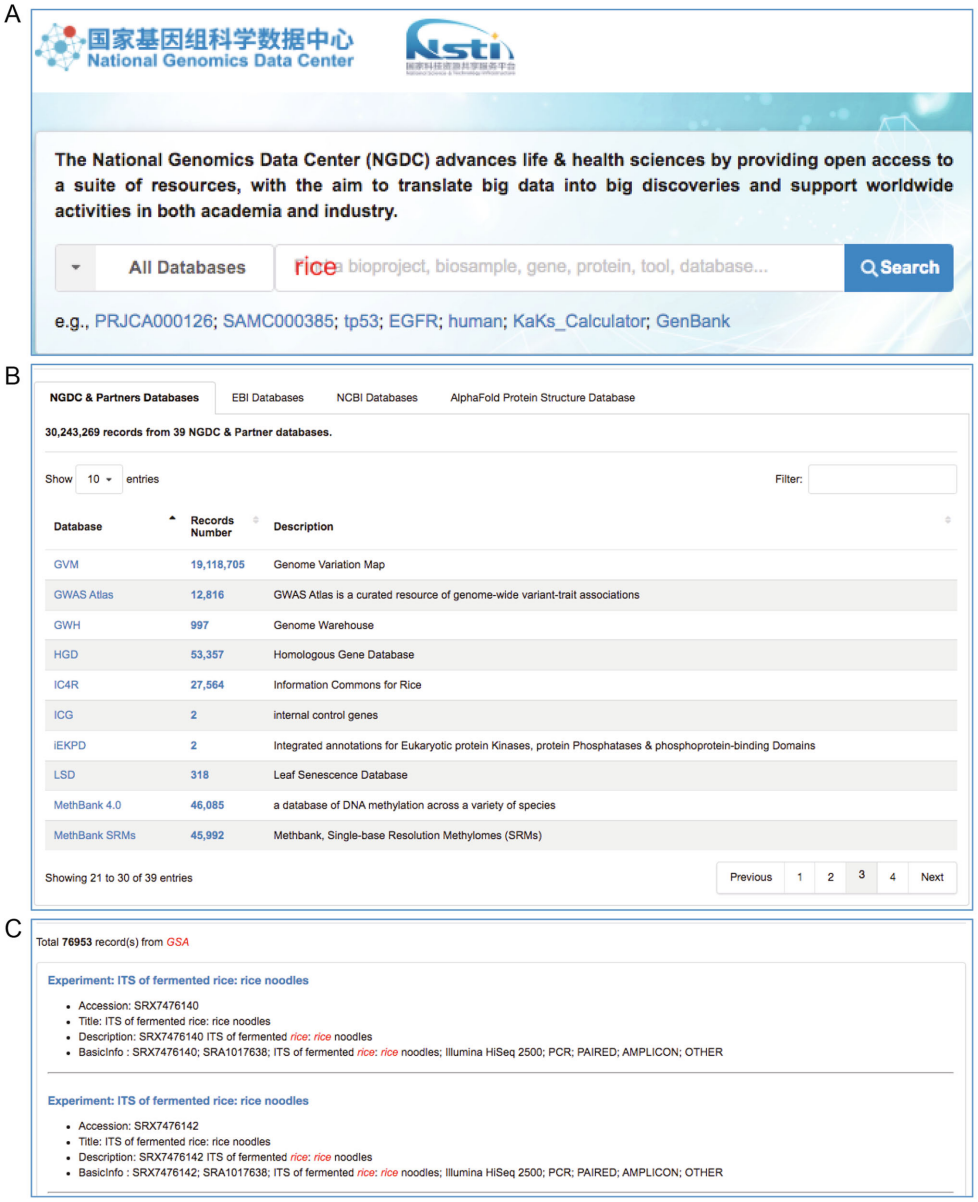
Screenshots of BIG-Search, including **A** search-bar and keyword input (e.g., rice), **B** summary of search results **C** datasets with of hyperlink of search results (e.g., GSA)

To better provide users with data retrieval and analysis services, we have developed a comprehensive search engine and a suite of application tools. The BIG Search (https://bigd.big.ac.cn/search) is a distributed and scalable full-text search engine for a large number of biological resources, providing one-stop, cross-database search services for the global research community. Currently, the BIG Search includes data indexes from all NGDC’s resources and 55 partner resources (see details at https://bigd.big.ac.cn/partners) as well as European Bioinformatics Institute (EBI) resources based on EBI Search RESTful API (Madeira et al. 2019), NCBI resources powered by NCBI Entrez (Gibney and Baxevanis 2011) and the AlphaFold Protein Structure Database (Jumper et al. 2021). As an example, shown in Fig. 2, BIG Search offers advanced search functions and cross-database search services for numerous data resources, providing users with a more convenient and efficient means of retrieving data.

While the Bioinformatics Toolkits (BiT, https://ngdc.cncb.ac.cn/bit), is a platform that integrates a great variety of tools that can be used for sequence alignment, composition analysis, RNA expression, epigenome analysis, haplotype network construction, and data visualization. Many tools are developed in-house, and several public tools are further developed with extended functionality. e.g., KaKs_Calculator is an in-house developed toolkit that is capable of calculating selective pressure on both coding and non-coding sequences (Zhang 2022), which has been widely applied in plant evolution or selection analysis or studies. For protein-coding sequences, it integrates several methods to calculate nonsynonymous (Ka) and synonymous (Ks) substitution rates. Particularly, it adopts model selection and model averaging to include as many features as needed for accurately capturing evolutionary information in protein-coding sequences. Similar to the Ka/Ks ratio for coding sequences, selection on non-coding sequences can be quantified as non-coding nucleotide substitution rate (Kn) normalized by synonymous substitution rate of adjacent coding sequences. The KaKs_Calculator 3.0 is implemented in standard C++ language, enabling higher efficiency and easy compilation on different operation systems (Linux/Windows/Mac). The package of KaKs_Calculator 3.0, including compiled executables, a Windows application with graphical user interface (GUI), source codes, and example data, accompanying with detailed instructions and documentation, is freely available for academic users at the CNCB-NGDC (https://ngdc.cncb.ac.cn/biocode/tools/BT000001). A graphical user interface demo is shown in Figure S1.

Overall, our BIG search engine is beneficial for quickly finding the desired data resources, while the BIT platform is particularly useful for end users who may not have a strong data analysis or computational background.

## Conclusion

In summary, NGDC provides a comprehensive web service for plant data management, including plant multi-omics data, functional knowledge and application tools. In the era of big data and the rise of artificial intelligence methods, these resources are expected to collect a wider range of omics data and apply deep learning techniques to analyze the intricate relationships between multidimensional omics data and agriculturally significant phenotypic traits, providing invaluable resources for plant researchers engaged in AI-driven breeding.

In the future, NGDC will continuously follow up the plant frontiers and enrich its plant data resource system. For example, pan-genome analysis and GWAS analysis of SVs have brought new perspectives and discoveries to plant research (He et al. 2023; Li et al. 2023; Liu et al. 2020). And new genomic technologies such as T2T, will make it more possible to explore the complex structure variations (SVs) of the plant genomes at the “species” level or even “genera” level with higher accuracy and sensitivity. It is valuable to construct a comprehensive data resources by collecting these high-quality assemblies, and integrating population structure, phylogeny, selection signals, and SV knowledge related to important traits. Furthermore, single-cell and spatial omics techniques have greatly increased the dimensions and precision of omics data, and help us to understand the characteristics and functions of individual cells and also support us to study the spatial distribution of gene expression at the tissue or single-cell level. Severalome resources in this field have been established, including PlantscRNAdb (http://ibi.zju.edu.cn/plantscrnadb/) (Chen et al. 2021a), PsctH (http://jinlab.hzau.edu.cn/PsctH/) (Xu et al. 2022), PCMDB (https://www.tobaccodb.org/pcmdb/homePage) (Jin et al. 2022), RCAR (http://www.elabcaas.cn/rcar/index.html), SODB (https://gene.ai.tencent.com/SpatialOmics/) (Yuan et al. 2023). Therefore, NGDC will continue to make efforts to provide a series of newly developed and integrated databases, making full use of these cutting-edge technologies and data, and paving the way for the implementation of genomic data in plant breeding.

Meanwhile, there are also many outstanding international plant data resources that are constantly updated and worth learning from, such as Gramene (https://www.gramene.org/) (Tello-Ruiz et al. 2016), TAIR (https://www.arabidopsis.org/index.jsp) (Lamesch et al. 2012), TRY (https://www.try-db.org/TryWeb/Home.php) (Kattge et al. 2020), Phytozome (https://phytozome-next.jgi.doe.gov/) (Goodstein et al. 2012), and all of them are curated in Database Commons (https://ngdc.cncb.ac.cn/databasecommons/) (Ma et al. 2022). However, currently, there is currently a lack of large-scale plant-related data resources or projects similar to The Cancer Genome Atlas Program (TCGA), the ENCODE (Encyclopedia of DNA elements) Project (Consortium 2004) and Human Cell Atlas (https://www.humancellatlas.org/) (Regev et al. 2017). Therefore, it is hoped that the field of plant research will also converge, similar to population-based studies, and NGDC will continuously track the advancements of cutting-edge plant science research, persistently updating and enhancing the existing data resource framework.

### Supplementary Information

Below is the link to the electronic supplementary material.

Supplementary Table S1. The top 50 species in CNCB-NGDC, ordered by data volume in GSA (DOCX 31 KB)

Supplementary Figure 1. Demo of KAKA-Calculator, including (A) graphical user interface, it contains two panels that are devised for CDS and NCS, respectively, (B) screenshots of input file, pair-alignment sequence data, (C) running parameter setting, and result (PDF 1305 KB)

## Data Availability

Data sharing is not applicable to this article as no datasets were generated or analyzed during the current study.
