# Supplementary material for: Plant genomic resources at National Genomics Data Center: assisting in data-driven breeding applications
Source: aBIOTECH. 2024 Feb 2;5(1):94–106. doi: 10.1007/s42994-023-00134-4 (PMC10987443; doi:10.1007/s42994-023-00134-4)
Supplement: Supplementary file 1 — Supplementary Table S1. The top 50 species in CNCB-NGDC, ordered by data volume in GSA (DOCX 31 KB) [file 42994_2023_134_MOESM1_ESM.docx]

**Table S1** The top 50 plant species

| **Class** | **Scientific name** | **Common name** | **GSA** | **GWH** | **GVM** | **GEN** | **Methbank** | **PlantPan** |
| --- | --- | --- | --- | --- | --- | --- | --- | --- |
| **Food crops** | *Oryza sativa* | Rice | **√** | **√** | **√** | **√** | **√** | **√** |
|  | *Triticum aestivum* | Bread wheat | **√** | **√** | **√** | **√** |  |  |
|  | *Zea mays* | Maize | **√** | **√** | **√** | **√** | **√** | **√** |
|  | *Triticum urartu* | - | √ | √ | √ |  |  |  |
|  | *Fagopyrum tataricum* | Tartarian buckwheat | √ | √ |  |  |  |  |
|  | *Solanum tuberosum L.* | Potato | √ | √ |  |  |  | √ |
|  | *Setaria italica* | Foxtail millet | √ | √ | **√** |  |  |  |
|  | *Hordeum vulgare* | Barley | √ | √ |  |  |  |  |
|  | *Sorghum bicolor* | Sorghum | **√** | **√** | **√** | **√** |  | **√** |
|  | *Secale cereale* | Rye | √ | √ | √ |  |  |  |
|  | *Oryza nivara* | Oryza sativa f. spontanea | √ | √ |  |  |  |  |
| **Cash crops** | *Glycine max* | Soybean | **√** | **√** | **√** | **√** | **√** | **√** |
|  | *Brassica napus* | Oilseed rape | **√** | **√** | **√** | **√** | **√** | **√** |
|  | *Solanum lycopersicum* | Tomato | **√** | **√** | **√** | **√** | **√** | **√** |
|  | *Vitis vinifera* | Grape | √ | √ | √ |  |  |  |
|  | *Camellia sinensis* | Tea | √ | √ |  |  |  |  |
|  | *Cucumis sativus* | Cucumber | √ | √ | √ |  |  |  |
|  | *Malus domestica* | Apple tree | √ | √ |  |  |  |  |
|  | *Cucumis melo* | Oriental melon | √ | √ |  |  |  |  |
|  | *Brassica rapa* | Field mustard | √ | √ | √ |  |  | √ |
|  | *Cucumis melo subsp. agrestis* | Cucumis callosus | √ | √ |  |  |  |  |
|  | *Gossypium hirsutum* | Cotton | √ | √ | √ | √ |  |  |
|  | *Sesamum indicum* | Koba | √ | √ |  |  |  |  |
|  | *Gossypium barbadense* | Egyptian cotton | √ | √ |  |  |  |  |
|  | *Vigna radiata* | Mung bean | √ | √ | **√** |  |  |  |
|  | *Prunus mira* | - | √ | √ |  |  |  |  |
|  | *Citrullus lanatus* | Wild melon | √ | √ |  |  |  |  |
|  | *Saccharum officinarum* | Noble cane | √ | √ |  |  |  |  |
|  | *Ipomoea batatas* | Sweet potato | √ | √ | **√** |  |  |  |
|  | *Manihot esculenta* | Cassava | √ | √ | √ |  | √ |  |
|  | *Hevea brasiliensis* | Para rubber tree | √ | √ | √ |  |  |  |
|  | *Phaseolus vulgaris* | Common bean | √ | √ | √ |  | √ |  |
|  | *Fragaria x ananassa* | Strawberry | √ | √ |  |  |  | √ |
|  | *Raphanus Sativus* | Radish | √ | √ |  |  |  | √ |
|  | *Vigna unguiculata* | Cowpea | √ | √ | √ |  |  |  |
|  | *Phoenix dactylifera* | Date palm | √ | √ | √ |  |  |  |
|  | *Daucus carota* | Carrot | √ | √ | √ |  |  |  |
|  | *Populus trichocarpa* | Poplar | √ | √ | √ |  |  |  |
|  | *Populus tomentosa* | Chinese white poplar | √ | √ |  |  |  |  |
|  | *Cunninghamia lanceolata* | China fir | √ | √ |  |  |  |  |
|  | *Populus euphratica* | Euphrates poplar | √ | √ |  |  |  |  |
|  | *Prunus mume* | Japanese apricot | √ | √ | √ |  |  |  |
| **Feed crops** | *Medicago sativa* | Sickle alfalfa | √ | √ |  |  |  |  |
|  | *Echinochloa crus-galli* | Barnyard grass | √ | √ |  |  |  |  |
| **Medicinal crops** | *Arabidopsis thaliana* | Thale cress | √ | √ |  | √ |  | √ |
|  | *Panax notoginseng* | Sanchi ginseng | √ | √ |  |  |  |  |
|  | *Capsicum annuum* | Pepper | √ | √ | √ |  |  |  |
|  | *Salvia miltiorrhiza* | Dan shen | √ | √ | √ |  |  |  |
|  | *Nicotiana tabacum* | Tobacco | √ | √ |  |  |  |  |
|  | *Cannabis sativa* | Cannabis | √ | √ | √ |  |  |  |
| **Total number of Species** | | | **1850** | **1926** | **31** | **9** | **7** | **11** |

Note: species are listed by data volume in GSA

**TABLE AND FIGURE LEGENDS**

**Supplementary Table S1.** The top 50 species in NGDC-CNCB, ordered by data volume in GSA.
