# Supplementary figures and images for: Plant genomic resources at National Genomics Data Center: assisting in data-driven breeding applications
Source: aBIOTECH. 2024 Feb 2;5(1):94–106. doi: 10.1007/s42994-023-00134-4 (PMC10987443; doi:10.1007/s42994-023-00134-4)

A

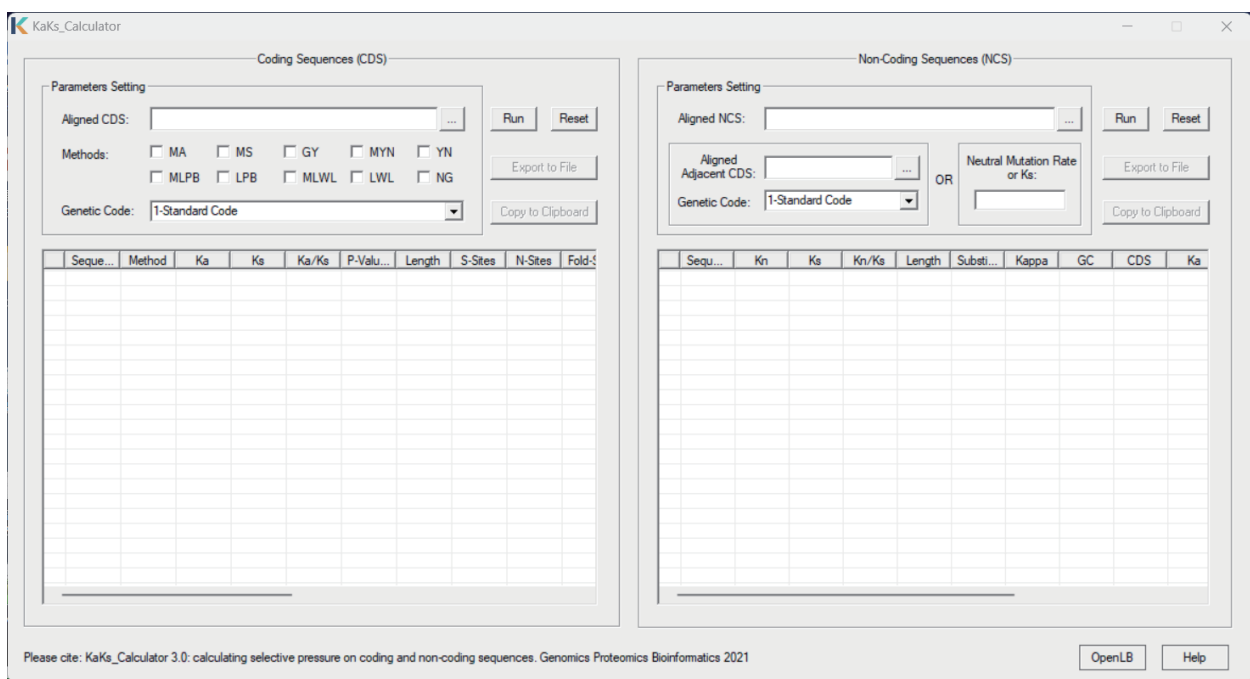

B

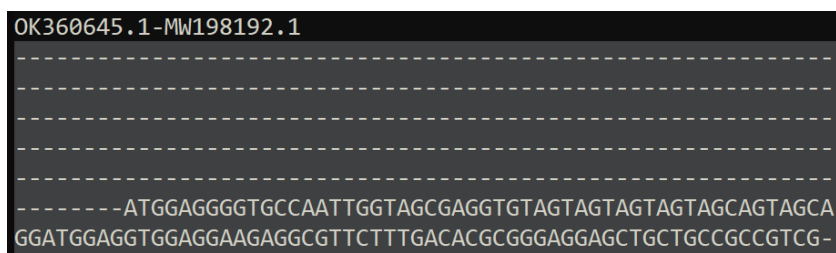

C

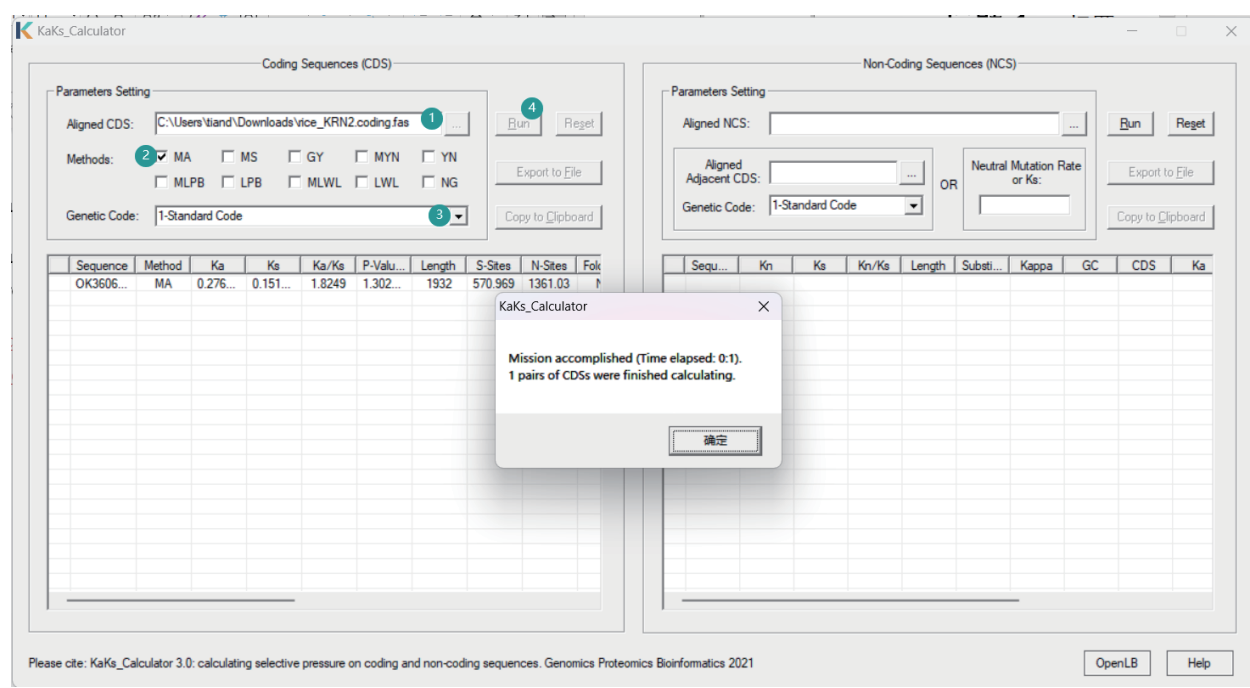

Supplement: Supplementary file 2 — Supplementary Figure 1. Demo of KAKA-Calculator, including (A) graphical user interface, it contains two panels that are devised for CDS and NCS, respectively, (B) screenshots of input file, pair-alignment sequence data, (C) running parameter setting, and result (PDF 1305 KB) [file 42994_2023_134_MOESM2_ESM.pdf]
